# Motivations for food prohibitions during pregnancy and their enforcement mechanisms in a rural Ghanaian district

**DOI:** 10.1186/s13002-015-0044-0

**Published:** 2015-07-17

**Authors:** Samson K. Arzoaquoi, Edward E. Essuman, Fred Y. Gbagbo, Eric Y. Tenkorang, Ireneous Soyiri, Amos K. Laar

**Affiliations:** Department of Population, Family, and Reproductive Health, School of Public Health, Box LG 13, Legon, Accra Ghana; Department of Biological Environmental and Occupational Health Sciences, School of Public Health, Box LG 13, Legon, Accra Ghana; Marie Stopes International, Accra, Ghana; Department of Sociology, Memorial University, St. John’s, NL A1C 5S7 Canada; South East Asia Community Observatory (SEACO) School of Medicine & Health Sciences Faculty of Medicine, Nursing & Health Sciences MONASH University, Bandar Sunway, Malaysia

**Keywords:** Food taboos, Food prohibitions, Pregnancy, Beliefs, Motivators traditional enforcement mechanisms, Ghana

## Abstract

**Background:**

Food taboos are known from virtually all human societies and pregnant women have often been targeted. We qualitatively assessed food taboos during pregnancy, its motivating factors, and enforcement mechanisms in the Upper Manya Krobo district of Ghana.

**Methods:**

This was an exploratory cross sectional study using qualitative focus group discussions (FGDs). Sixteen FGDs were conducted. Participants were purposively selected using the maximum variation sampling technique. Tape recorded FGDs were transcribed verbatim and analyzed using Malterudian systematic text condensation technique.

**Results:**

All the participants were aware of the existence of food prohibitions and beliefs targeting pregnant women in Upper Manya Krobo. The study identified snails, rats, hot foods, and animal lungs as tabooed during pregnancy. Adherence motivators included expectation of safe and timely delivery, avoidance of “monkey babies” (deformed babies); respect for ancestors, parents, and community elders. Enforcement mechanisms identified included constant reminders by parents, family members and significant others. Stigmatization and community sanctions are deployed sparingly.

**Conclusions:**

Food taboos and traditional beliefs targeting pregnant women exist in Upper Manya Krobo. Pregnant women are forbidden from eating snails, rats, snakes, hot foods and animal lungs. To a large extent, socio-cultural, and to a lesser, health concerns motivate the practice.

## Background

Food taboos are known from virtually all human societies [1–5]. They form a codified set of rules about which foods or combinations of foods may not be eaten. The origins of these prohibitions are as varied as their motivators and govern particular phases of the human life cycle. These prohibitions are sometimes associated with special events such as menstrual period, pregnancy, childbirth, lactation, and – in traditional societies – preparation for the hunt, battle, wedding, funeral, et cetera.

Of interest to this paper, are food prohibitions during pregnancy given their implications for both maternal and fetal health. Adequate supply of nutrients is required to maintain a delicate balance between the needs of the mother and those of the fetus. Inadequate supply will cause a state of biological competition between the mother and the conceptus in which the well-being of both is at serious risk [6]. To meet the increased nutrition needs, healthful eating is of utmost importance during pregnancy and lactation. All societies have traditional beliefs regarding harmful and beneficial foods for women during pregnancy [7], and are transmitted and sustained by a combination of familial and cultural modes [8]. These prohibitions usually do not conform to the modern biomedical notion about the proper types and amount of foods needed by pregnant women to safeguard optimal materno-fetal nutrition.

Although food prohibitions during pregnancy are common in Africa, the forbidden foods vary by setting. A study among pregnant women in Tanzania enumerated various forms of food taboos ranging from eating fish which was believed to hurt the mother’s abdomen and cause late delivery, and eating of farm animals perceived to make babies pick up characteristics of the farm animals [9]. It is noteworthy that some food taboos may be helpful during pregnancy. The American College of Obstetricians and Gynecologists notes that pregnant women should avoid eating shark, swordfish, king mackerel, or tilefish because they contain high levels of mercury that can be harmful to the developing fetus [10]. In Ghana, women are expected to avoid certain foods when pregnant in certain cultures out of fear and belief that these could harm unborn children [11]. Some other Ghanaian dietary taboos are that pregnant women are not expected to eat snail to avoid giving birth to drooling babies and children. Among the Kassena and Nankana of the Upper East Region, pregnant women are restricted to vegetarian diet; they must not eat meat and groundnut as this could lead to the birth of 'spirit children’ (children deemed to possess spirits). In her study among the Akwapims, [12] observed that expectant women were forbidden to buy tomatoes, pepper, okra and eggplant from the market. If they did, it was believed that their children will be infected with severe rashes and will consequently suffer from some form of disability. Children on the other hand are prohibited from eating egg. Proponents argue that giving eggs to children is associated with thievery when they grow up [9, 13]. Similar taboos and restrictions have been found among the people of Anyamtan in the Dangme West District. Other local justifications (mainly from folkloric sources) exist in support of prohibitions of snails, okra, ripe plantain, and coconuts. Snails and okra are perceived to cause the baby to slime, while ripe plantain and pineapple are said to cause waist pain, early labour or abortion. Coconuts on the other hand are believed could make a baby blind, a condition described as "white eye [12].

While acknowledging the above, and other studies on the subject in Ghana [14–17], it must be noted that the various social, cultural, and linguistic groupings in Ghana might have different food taboos, affecting vulnerable populations such as children, and pregnant women. Knowledge about these group-specific practices are relevant for successful public health interventions in communities where such practices are common.

The nutritional hazards and health implications of food taboos and preferences have been extensively discussed [1–3]. When practiced in pregnancy, adverse consequences such as depletion of vital nutrients required by the mother and the unborn are most likely. Most of the tabooed foods are key sources of protein. Protein, the nutrient, provides cell-building tasks for the growing baby, especially in brain development. As shown by the literature presented above, high caloric foods, foods rich in vitamins and minerals such as banana, snails and peanut are equally forbidden. Such foods play critical roles in the promoting, and preserving health throughout the various phases of life. Brito and Estacio’s recent work clarifies the effect of food taboos including on prenatal nutrition. To our knowledge, neither the extent of the practices of food prohibitions in pregnancy in the Upper Manya Krobo, nor the health implications of the practice has been done.

While food taboos have deleterious consequences for maternal and child health outcomes, such taboos and the motivations behind them have rarely been documented in the literature. Using qualitative data from a rural Ghanaian district, we contribute to the existing but scant body of literature by documenting these taboos and the motivators for such practices. Further, the study analyzes the traditional mechanisms for transmitting and enforcing food taboos.

## Methods

### Study type, population sampling and summary of field procedures

This was an exploratory cross sectional study using qualitative methods. The study involved Ghanaian men and women from diverse backgrounds, and traditional leaders in six communities in the Upper Manya Krobo District, which is one of the twenty-one districts in the Eastern Region of the Republic of Ghana.

A total of 155 participants were selected to represent six study communities or sub-districts. The 155 participants included 46 pregnant women, 30 elderly women, 42 elderly men and 17 women of reproductive age. The participants were purposively selected using the maximum variation technique. This sampling strategy implies an intentional and systematic selection of study participants with varying characteristics with reference to the topic of concern. In this regard, the composition of the study participants was diverse with regard to socioeconomic status, ethnicity, age, pregnancy status, gestational age, and sex. Sex-specific groupings were done to ensure that males do not influence the discussions.

In total we had focus group discussions (FGDs) and key informant interviews (KIIs) with 155 respondents. A total of ten focus groups consisting of an average of eight participants were organized across six communities in the district Four (4) FGDs (two male older and younger men) groups and two female (older and younger women) groups) and eight key informant interviews with healthcare providers were utilized. These assessed knowledge, current participation, desire to participate, improvement of male participation, beliefs in involving males during pregnancy, delivery and after childbirth.

Teams of three researchers made two weeks of daily visit to each of the six study communities. After explaining the purpose of the visit to the Assembly Member, district elders, queen mothers, and community elders in the district headquarter, a unanimous permission was granted, total cooperation and support offered. Of note, the Assembly Member is an elected community member by the National Constitutional act (Act 426) who acts as the liaison between the District Assembly and the community and s/he who plays a critical role in community development by virtue of the position [18].

Additional meetings were also held at every study site involving the Assembly Member to seek permission and obtain informed consent for participation of the community member in the FGDs. Four data collectors were recruited, trained and served as interviewers to conduct the focus group discussions while the key informant interviews was conducted by the first author. All the FGDs were tape recorded and hand written notes taken with the permission of the study participants at all the study sites.

### Data processing and analysis

The tape recorded FGDs and KIIs supported by the handwritten field notes were transcribed and where applicable translated from *Krobo* to English. Analysis were manually using the principles of systematic text condensation as described by Malterud (2001). This entails four steps: repeated review of the transcript to gain thorough sense of the overall content in the texts, identifying central meaningful units in the material, condensation of the content through a coding of the text, and finally creating categories that contain the condensed meaning of the main themes in the material [19]. Sections of the discussions were quoted verbatim, and some modified to enhance readability. We were aware that manual analysis of the data could result in the introduction of personal idiosyncrasies into themes. Therefore themes from the manual analysis were later validated by NVivo qualitative data analysis software (QSR International Pty Ltd. Version 9, 2010).

### Ethical considerations

In line with national research standards, ethical approval was obtained from Ghana Health Service Ethical Review Committee. Permission for the conduct of the study was sought and obtained from the local government representatives (The Assembly Member of the selected sub- districts), community leaders, and queen mothers. The right of the people to participate in the study and to opt out without any precondition at any time was explained and respected. The purpose of the study was explained to all and the participants signed/endorsed the consent form before the conduct of every FGD. Participation in the study was thus, completely voluntary, no financial or material benefits were given, although light refreshment was served at the end of every FGD. The participants, who were all adults further consented to the publishing of the study findings. The privacy and confidentiality of every participant was ensured throughout the study period. Identity numbers were used to disguise the discussants’ identity. Every member of the data collection and analysis team were cautioned during the training process to maintain straight confidentiality and anonymity throughout the study.

## Results and discussion

A total of one hundred and fifty-five (155) respondents comprising 46 pregnant women, 30 elderly women, 42 elderly men and 17 Women in Fertility Age (WIFA) participated in the study (Table 1). Themes that emerged from our interactions with the participants and key informants are presented and discussed.

**Table 1:** Demographic Characteristic of study participants

Variables	Number of Participants
Number of Children	*n = (155)*
None	9
One child	8
Two children	18
Three children	30
Four children	23
Five children and Above	67
Age	*n = (155)*
45 and over	51
40 -44	9
35-39	38
30-34	18
25-29	26
20-24	8
18-19	5
Educational Background	*n = (155)*
No Education	18
Junior High School (JHS)	112
Senior High School (SHS)	24
Tertiary	1
Religion	*n = (155)*
Pentecostal	59
Christ Apostolic Church (CAC)	68
Presbyterian	18
Roman Catholic	2
Methodist	4
Muslim	4

### Food items tabooed during pregnancy

All participants admitted being knowledgeable about various taboos during pregnancy, labor, after birth and enumerated the common taboos (Table 2). Some discussants during the FGDs explained food taboo as:

**Table 2:** Traditional Beliefs during Pregnancy

Pregnant women should not sit at the same place for long after eating
Pregnant women are not to take bath at night.
Pregnant women should not keep bath-water long before bathing.
Pregnant women should never cross their legs whiles sitting
Pregnant women should not carry a whole bunch of palm nuts, plantain and banana
Pregnant women should not eat at night.
Pregnant women should not eat hot food
Pregnant women should not cut firewood.
Pregnant women should not split firewood.
Pregnant women should not lie on their back while eating.
Pregnant women should not respond to calls at nights
Pregnant women should not leave their hair open
Pregnant women should always conceal their food from strangers
Pregnant women should never have sex with another man
Pregnant women should never steal
Pregnant women should not eat openly in the public
Pregnant women should not reject food or gift from her husband and family members
Pregnant women should not use two different colors of sandals

*“All the laws as instituted by our people about foods that we are not to eat or touch” (66 year old woman in Somanya).*

*“Food that you are not supposed to touch or eat” (22 year old, Okotokrom)*

*“Food that does not go with our culture to eat or drink” (31 year old, Okotokrom)*

*“Foods when you eat can harm you or cause problems for the community” (24 year pregnant woman, Nkuranka).*

The study revealed, rats, snails, snake, hot food and animal lungs as prohibited foods during pregnancy. Similar studies conducted in rural Northern Ghana, Dove [20] mentioned that in addition to herbal remedies, pregnant women were taught about taboos by their immediate families, extended families, and communities. Other tabooed foods and their perceived effects were identified by Dove (ibid) are as follows:

- Honey causes respiratory problems for the child at birth.
- Bambara beans cause respiratory and skin problems for the child at birth.
- Corn flour is linked to heavy bleeding at delivery.
- Shea butter can cause difficulty in delivery.
- Eggs, fresh meat, fresh milk, and cold and sugary foods make the unborn baby large, contributing to a difficult delivery and possible death of the mother.

Another Ghanaian study, revealed that pregnant women in some Ghanaian communities avoid fufu, gari, kokonte (all cassava based foods), fresh fish, corn dough porridge, eggs, banana, crabs and ripe plantain [21]. Findings from this current study support others in which food taboos during pregnancy are found to be more elaborate, nutritionally significant and differ only in type and characteristics [22, 23].

Globally, pregnant women hold onto certain food taboos to ensure healthy babies [24].’ For instance, in most Western cultures, rats and mice are considered either unclean vermin or pets that carry plague hence unfit for food. However, rats are commonly eaten in rural Thailand, Vietnam and other parts of Indochina [24]. This appeared contradictory to the current study finding since, all the participants in FGD unanimously declared that rat is a food taboo during pregnancy*. Some respondents indicated that:*

*“When our forefathers first came to settle on this land, they settle on top of the mountains. They could not dig graves to bury their dead because of the rocky nature of the ground but instead used caves between rocks to bury. It was later discovered that rats would enter and dig out the bones of their dead relatives. That is the main reason why we do not eat rats because we believe that they burrow into graves and feed on dead bodies as well.”* (*A 46 years Man from Somanya).*

Data collected from all FGDs in Somanya, Apalau and Okotokrom indicate that snakes are not commonly eaten in many parts of Ghana. Although, all participants indicated that snake meat is generally avoided, participants in Somanya, Apalau and Okotokrom, view snake meat as a taboo during pregnancy thus, denying pregnant mothers the opportunity to decide whether to eat or not. Participants believe that babies born to mothers who eat snake meat during pregnancy will eventually develop dry scaly skin after birth. While on the contrary participants in Nkuranka, Okornya and Ponponya view this avoidance as a mere choice or dislike.

A respondent during the FGD indicated that: *Although there is no tangible cultural association with health related reason for avoiding snake meat, it is regarded a dishonorable meat and modernization may have played a key role in the avoidance of this meat (25 year old woman from Somanya).*

Another respondent reported:

*“It is rare to see someone eating snake in Ghana. It is not a taboo but majority of Ghanaians don’t like snake. Because this has been happening for long time now, some people view it is as a taboo.” (A 72 years old man from Okotokrom)*

Kruger and Gericke (2003) made similar observation that although mere avoidance of potential food does not in itself signify a food taboo, it is easy to see how regular avoidance can turn into a tradition and eventually end up as a food taboo [25].

This observation by Kruger and Gericke (2003) was partly reflected in our study where some respondents indicated their dislike for certain foods.

*“We the Krobos don’t like snails. We are not even allowed to touch it. Strangers are also not allowed to bring it in our homes or eat it using our utensils. Snails treat us badly and that is why pregnant women are not supposed to eat or touch because it will also affect the baby in the stomach”. (An elderly man in Somanya)*

Another respondent explained:

*Skin eruptions and generalized life threatening skin rashes in both the mother and baby are commonly attributed to those who eat snail during pregnancy (18 year old Krobo man).*

Although an unpublished work by Jacob P. Anankware presents the potential of neglected and underutilized insect species for nutrition and health in Ghana, with resistance in some communities, the practice entomophagy (consumption of insects as food) did not appear to be prohibited during pregnancy in Upper Manya Krobo district. It is known that insects particularly termites and the African palm weevil (Rhychophorus phoenicis) larva are a delicacy to some tribes/groups in palm wine-tapping communities in Ghana (Jacob P. Anankware, Personal Communications).

As noted earlier, snails’ prohibition as food has been motivated by its association with slimy salivation and dripping mouth of a baby whose mother ate snail during pregnancy. Also in our studied area, animal lungs are taboo for men due to its perceived association with asthma. Although this is one of the male-specific food taboos, pregnant women are also affected since it is impossible for them to determine the sex of their babies before delivery*. A respondent stated in the FGD that:*

*It is strongly believed that a pregnant woman carrying a male fetus who eats animal lung will eventually have a child diseased with asthma (45 year old woman from Somanya).*

Another respondent stated that:

“*Lungs of animals are food taboos for men only because it can cause asthma in men who eat them hence, pregnant women are not allowed to eat lungs. It is strongly believed that if the mother is carrying a male fetus, that child will eventually develop, after birth.” (A 34 year old woman from Nkuranka)*

This findings may not be unique to pregnant women in this rural Ghanaian district. Indeed, Myaruhucha (2009) had asserted that, cultural food restriction during pregnancy is a common practice in developing countries. In one of the communities in Nigeria, for example, it was found that about 66 % of women avoided milk [26] while in another village; Ebomoyi [27] observed that practically all pregnant women avoided meat (98 %). In Sudan, a study by Boucher revealed that fatty foods were abstained from by a sizeable proportion of pregnant women [28].

### Motivators

Respondents’ reasons for obeying food taboos in this study were grouped into three broad categories: health reasons, respect for the ancestors and respect for significant others.

Health related reasons include; Safe and timely delivery, Avoidance of ‘monkey-babies” (deformed babies), Healthy baby and mother, and avoidance of epidemic diseases. A pregnant woman explained why she adheres to the taboos as follows:

*“This is the only way I will deliver safely and my baby will have no problem” (A 32 years old pregnant woman, Nkuranka).*

Another respondent indicated that:

*“If I don’t observe food taboos, spirits and witchcrafts will destroy my pregnancy” (A 29 years old pregnant woman, Okotokrom).*

*Another respondent explained:*

*“When we don’t obey our forefathers…, they get annoyed with us and bring sickness or hard time (misfortunes) to the community. If you plant anything for example the crops will not grow well and hunger will fall on us” (An elderly man, Somanya)*

Most parents and community elders/leaders are usually keen about the health and safety of the children and community members. Disobedient children/community members are constantly frowned on. In extreme cases or serious offences, they are excommunicated from the family or the community. To guard against these odds, pregnant women exhibit high levels of respect for their parents and community elders. A respondent said *“Our people will be happy with you if you listen to their advice. If you don’t listen anything which will happen to you, they will say you are responsible” (24 years old, woman, Okornya).*

There is strong evidence from all the FGDs conducted that all those adhering to the taboos understand the reasons for doing so. These restrictions as observed are associated with positive antenatal health behaviors and positive pregnancy outcomes which motivate pregnant women to observe the taboos [29].

#### Food taboos and beliefs for health reasons

Food taboos are particularly regarded by people of Upper Manya as a form of instruction or command from God passed down generations to safeguard them against evil and diseases. Challenging these taboos is considered not only as blasphemous but a health risk.

Timely and safe delivery is regarded as one that is done at home, assisted by a traditional birth attendant (TBA) or by experienced community members. Hospital delivery is thus seen as failure in the normal delivery process and punishment.

A respondent said:

*Those who don’t observe food taboos in pregnancy have complicated delivery including caesarean section (30 years old woman Nkuanka).*

*Another respondent stated:*

*I am not supposed to eat snails because I know this is the only way I will deliver safely a healthy baby” (A 32 years old pregnant woman, Nkuranka).*

Congenital malformations are wildly regarded as punishment for disobedience of cultural norms including eating prohibited foods and the disregard for beliefs for protection and safety of the pregnancy. Two members of the FGD conducted in Nkuranka said,

“*If pregnant woman continue to take bath at night, she will one day take bath with evil spirits. If you take bath with evil spirits, they will harm the baby and you deliver monkey baby” [*deformed baby].

*“If you eat the tabooed foods like snail, you will get sick. So many things [skin rashes] will come on your skin or you will deliver sick baby and sometimes the baby may ‘go back’ [die]. The foods the old people are talking about is to help us not to fall sick during pregnancy (A 22 years old pregnant mother, Nkuranka).*

All the participants agree that the surest way to ensure healthy pregnancy and deliver healthy baby is to obey the food taboos and adhere to the traditional beliefs about pregnancy.

*“If I do not respect the taboos and the laws, the spirits and witchcrafts will destroy the pregnancy or I deliver, the baby may get sick and die” (A 29 years old woman, Okotokrom).*

Martin [30] his study on food restrictions in pregnancy among mothers in Hong Kong reported that, in order to maintain harmony within the body (interpreted as a good state of health), pregnant women should avoid eating “wet-hot foods” (e.g., shrimp, mango, lychee, longan, and pineapple); as doing so will produce a “poisonous” energy which will manifest itself as allergic reactions or skin eruptions in the baby. Some of the participants in the current study communicated the community’s perceived association between food taboos and ill-health during pregnancy and childbirth.

A lamenting 34 year old woman in Okotokrom said,

*When you get sick and you are not getting well soon, your husband and his people will always say you eat something you are not supposed to eat or that you have done something wrong and you need to confess. If the worst thing happen, you deliver with monkey baby, they will condemn you and if you are not careful, your husband will leave you”*

It was gathered from the FGD in Apalau that one way ancestors can sanction is through disease outbreak that may affect an entire community. A woman reported that:

“*Sometimes when the spirits of our forefathers are vexed with the kind of things the young people are doing now, eating everything, pregnant doing anything she feels like, they can send sickness on everybody*” (a 72 years old woman).

#### Food taboos regarding respect for the ancestors

Respect for ancestral laws and guidelines for Krobos is cardinal. Many believe that disobedience of ancestral laws will lead to anger of the ancestors who may ruin havoc on either the individual or the community as a whole. A participant durng the FGD said:

*”When we do not listen to what our forefathers instruct us, sometimes they get annoyed with us and bring sickness or hard time to the community. If you plant anything for example the crops will not grow well and hunger will fall on us” (An elderly man, Somanya).*

Bolton [31] his study of food taboos among the Orang Asli in West Malaysia had noted that the Orang AsliTemiar practice food taboos and avoidances to maintain harmony with entities, natural and supernatural, and to prevent any misfortune or calamity from happening. This compares with some food taboos and restrictions that are most commonly practiced in relation to pregnancy in Africa [32]. Indeed more recent reviews and reports of food taboos among the Orang Asli in Peninsula Malaysia [33] share some similarities with food taboos in Africa in terms of their diversity and intent or purpose.

#### Food taboos as a symbol of respect for parents and community elders

Haslam, Lawrence, & Haefeli [29] noted that, theoretically, pregnancy restrictions may be seen as having both positive and negative impacts on health-related quality of life. Participants from our study agree that although some of the reasons for food taboos are trivial and sometimes based on mere speculations, they uphold them for fear of their perceived repercussions. The spirits of the forefather are believed to be residing very closely with the living ensuring that the genealogical values are upheld. A participant explained that breaking taboos and beliefs humiliates the ancestors who may vent out their anger in many deleterious ways from ill health, poor farms yields to death and in some cases collective punishment. Perceiving themselves as more vulnerable, pregnant women present themselves to be seen as utterly acceding. Manyande & Grabowska [34] on the other hand have argued that the question of whether or not these traditional practices protect women’s health during pregnancy has yet to be answered. Current evidence on the utility or disutility of the practice during pregnancy is not conclusive. There is a possibility that such practices during pregnancy could have both therapeutic and harmful consequences.

#### Food taboos as a factor in group-cohesion and group-identity

It should also be pointed out that the participants in FGDs conducted in Somanya, Apalau and Okotokrom expressed strong delight and pride in belonging to what they viewed as a unique cultural setting. The majority of these participants indicate the allegiance to their community and cultural values. To be regarded as a Krobo, they think, you must abide by what is said and done by the people of Krobo land. A respondent stated that:

“*As a Krobo woman, I have to avoid snail and all other things that I am not supposed to do as a Krobo. All over Ghana, we are known as people who don’t eat snail so, I cannot be a Krobo and eat snail…never” (an elderly woman, Okornya).*

*Another respondent stated: “Whether what they say is true or not, I don’tt know. But, once I am Krobo, must do what the Krobo culture is saying or else I will not be regarded as being part of the community. My own people will avoid me and even drive me away (*34 year old woman, Okotokrom)

Closely associated with this finding is an assertion made by Meyer-Rochow VB *(1998)* while looking at further reasons for food taboo adherence [23]. He mentioned that, any food taboo, acknowledged by a particular group of people as part of its ways, aids in the cohesion of this group, helps that particular group maintain its identity in the face of others, and therefore creates a feeling of "belonging". Thus, food taboos can strengthen the confidence of a group by functioning as a demonstration of the uniqueness of the group in the face of others. Food taboos and food habits can persist for a very long time and can be (and have been) made use of in identifying cultural and historical relationships between human populations.

### Enforcement mechanisms

The various Food Taboos and beliefs are enforced by constant reminders by various people. Every pregnant woman will be properly educated about the taboo whenever she attempts to break it. It was reported that:

*“When my mother sees me trying to eat something that I am not supposed to eat, she will quickly stop me” (30 years pregnant woman, Somanya)*

*“The community people too can always stop you if they see you trying to eat what you are not supposed to eat” (23 year woman, Okotokrom)*

*“My friends can also tell me not to eat certain foods that I am not supposed to eat according to our Krobo taboo” (27 years woman, Nkuranka)*

*“My husband also tells me not to eat the food our people forbids” (36 years woman, Okornya)*

During FGDs at all the study sites the participants noted that pregnant women who are noticed as disobedient to the taboo are advised repeatedly either by the husband, community elders or parents. In some cases, when the offence is considered grave and threatening, the pregnant woman is taken to a priestess (“Se wonne”) for Purification of the Womb (“musuyi”) to ensure safe delivery.

All the female participants at the six study sites said that their mothers were the ones who constantly reminded them about food taboos. On the contrary majority of the male participants indicated that they advised their wives about the dangers associated with disobeying the food taboos and beliefs. Moreover, the men admitted that when a pregnant wife constantly disregards the taboos and beliefs, she is often sent to her mother for further counseling. Most of the participants view traditional beliefs on food taboos as crucial during pregnancy. The teachings at all levels emphasize the dangerous consequences of breaking the taboos and traditional beliefs.

Most respondents suggested that, severity of the punishment was related to the taboo or belief when broken. Death of the mother, baby or both is the most severe form of punishment. Those delivering the punishment are: ancestors, spirits, and family heads or community elders. It is believed that power and authority used by those implementing the punishment come from God though he may not be directly involved.

A 22 year old pregnant woman at Nkuranka said:

*“If something happen to you or your baby, you do not know who is punishing you but we know that it is God who is doing the punishment because we don’t see him. Sometimes I also think it the work of evil spirits who are agents of the devil and not God. But whatever the case is, it is better to be on the safe side.”*

Another respondent stated:

“Every *woman in this community know the importance respecting taboos during pregnancy. That is why every girl in this district must undergo* Puberty rites (*Dipo) to become pregnant. (68 years old man from Somanya)*

We recognize that a discussion on the extent to which these food restrictions affect the nutritional status of adherers, falls outside the scope of this current qualitative study. We nevertheless situate our discussion on our appreciation of the nutrient content of the various forbidden foods, and nutrient requirements of the pregnant women. Among these rural dwellers, most of the forbidden foods, which have both high caloric and protein content, may contribute significantly to the total energy requirements of the affected population (pregnant women). Ojofeitimiet et al. [26] and Bolton [31] presented an extensive discussion on the nutritional hazards and health implications of food taboos and preferences in Mid-West Nigeria and West Malaysia respectively. In the current study, the majority of the taboos affect foods of animal origin. Brandon and Rupe [10] have recently enumerated **t**he value of proteinaceous foods in pregnancy. When protein and other vital nutrients such as vitamins, minerals, and lipid-containing foods are deprived in pregnancy, adverse consequences such as depletion of these vital nutrients required by the mother and the unborn are most likely.

Our paper shows that high caloric foods, and foods rich in vitamins and minerals are equally forbidden. Such foods play critical roles in the promoting, and preserving health throughout the various phases of life – particular during pregnancy. Emblematic of the pregnant woman in this peasant farming community, uninterrupted overwork and child bearing is a norm. These women who often perform much of the manual labor within the village, including the cultivation and production of food, compounded by the nutritional strain of reproduction are vulnerable to malnutrition. Prohibitions from consumption of important nutrient sources may exacerbate this already precarious status of these women. We argue therefore that food prohibitions particularly during pregnancy in this setting is worthy of recognition as a public health problem that needs to be addressed. We hope that the naturalness with which the average Ghanaian ignores this problem will be destabilized by the publishing, and publicizing of these findings. Given that majority of those who engage in the practices may be genuinely ill-informed about food matters, we suggest an educational or health intervention that is sympathetic, and not the current paternalistic model of Ghanaian healthcare delivery system. To be able to deliver sound and acceptable nutrition education, nutrition training establishments, as well as medical and paramedical training establishments need to address some of these concerns in their pre-service training curricular. Conscious efforts should be made at the delivery phase, to promote good traditional practices or even health neutral ones may motivate community to buy-in into these interventions,

This study confirms the existence of food taboos and traditional beliefs targeting pregnant women in a rural Ghanaian district. Pregnant women are forbidden from eating various foods including snails, rats, snakes, hot foods and animal lungs. To a large extent, socio-cultural, and to a lesser, health concerns motivate the practice. These practices may have negative health implications on the women. We suggest that the lay public should be educated on these matters by designated health personnel, who themselves must first be given the needed capacitation.

## Abbreviations

WIFA: Women in fertility age
FGD: Focus group discussion

## References

[1] Brito Junior LC, Estacio AG (2013). [Food taboos in medicine: a hypothesis for pathophysiology regarding harmful food]. Rev Assoc Med Bras.

[2] Meyer-Rochow VB (2009). Food taboos: their origins and purposes. J Ethnobiol Ethnomed.

[3] Santos-Torres MI, Vasquez-Garibay E (2003). Food taboos among nursing mothers of Mexico. J Health Popul Nutr.

[4] Trant H (1954). Food taboos in East Africa. Lancet.

[5] Trigo M, Roncada MJ, Stewien GT, Pereira IM (1989). [Food taboos in the northern region of Brazil]. Rev Saude Publica.

[6] Wahid MA, Fathi SA (1987). Nutrition and the unborn baby. Ric Clin Lab.

[7] Mukhopadhyay S, Sarkar A (2009). Pregnancy-related food habits among women of rural Sikkim, India. Public Health Nutr.

[8] Patil R, Mittal A, Raghavia M (2010). Taboos and misconceptions about food during pregnancy among rural population of Pondicherry. Calicut Med J.

[9] Lozoff B, Klein NK, Nelson EC, McClish DK, Manuel M, Chacon ME (1998). Behavior of infants with iron-deficiency anemia. Child Dev.

[10] Brandon B, Rupe H: The everything guide to pregnancy nutrition and health: from preconception to post-delivery, all you need to know about pregnancy nutrition, fitness, and diet! Avon, Mass.: Adams Media Corporation; 2013. http://catalog.douglascountylibraries.org/Record/1151269.

[11] Boyacioglu AO, Turkmen A (2008). Social and cultural dimensions of pregnancy and childbirth in eastern Turkey. Cult Health Sex.

[12] Darko MO: Taboos on Women: A Case Study of Akwapim Traditional Area. *Unpublished BA dissertation*. Accra: Department of Sociology, University of Ghana; 1992.

[13] Lang A, Lee S (2014). Individual differences in trait motivational reactivity influence children and adolescents' responses to pictures of taboo products. J Health Commun.

[14] King S (2003). Maternal Mortality in Ghana: The Other Side. Res Rev.

[15] Amegah AK, Damptey OK, Sarpong GA, Duah E, Vervoorn DJ, Jaakkola JJ (2013). Malaria infection, poor nutrition and indoor air pollution mediate socioeconomic differences in adverse pregnancy outcomes in Cape Coast, Ghana. PloS one.

[16] Saaka M, Galaa S (2011). Improving the utilization of health and nutrition services: experience from the Catholic Relief Services supported the Development Assistance Programme in Ghana. Primary health care research & development.

[17] Brugha R, Kevany J (1994). Determinants of nutrition status among children in the eastern region of Ghana. J Trop Pediatr.

[18] Friedrich-Ebert-Stiftung F, IoLGS (2010). A Guide to District Assemblies in Ghana.

[19] Malterud K (2001). Qualitative research: standards, challenges, and guidelines. Lancet.

[20] Dove N (2010). A Return to Traditional Health Care Practices A Ghanaian Study. J Black Stud.

[21] Nti CA, Larweh PM, Gyemfua‐Yeboah Y (2002). Food consumption patterns, dietary quality and health status of expectant mothers: case studies in suburban and rural communities in Ghana. Int J Consum Stud.

[22] Fishman C, Evans R, Jenks E (1988). Warm bodies, cool milk: Conflicts in post partum food choice for Indochinese women in California. Soc Sci Med.

[23] Meyer-Rochow VB (2009). Journal of Ethnobiology and Ethnomedicine. J Ethnobiol Ethnomed.

[24] Goodburn EA, Gazi R, Chowdhury M. Beliefs and practices regarding delivery and postpartum maternal morbidity in rural Bangladesh. Stud Fam Plann. 1995;22–32. 7785065

[25] Kruger R, Gericke G (2003). A qualitative exploration of rural feeding and weaning practices, knowledge and attitudes on nutrition. Public Health Nutr.

[26] Ojofeitimi E, Elegbe I, Babafemi J (1982). Diet restriction by pregnant women in Nigeria. Int J Gynecol Obstet.

[27] Ebomoyi E (1988). Nutritional beliefs among rural Nigerian mothers. Ecol Food Nutr.

[28] Bouchier V (1984). Maternity care in the Sudd, southern Sudan. Trop Doct.

[29] Haslam C, Lawrence W, Haefeli K (2003). Intention to breastfeed and other important health-related behaviour and beliefs during pregnancy. Fam Pract.

[30] Martin D. Food restrictions in pregnancy among Hong Kong mothers. Changing Chinese Foodways in Asia. 2001;97–122.

[31] Bolton JM (1972). Food taboos among the Orang Asli in West Malaysia: a potential nutritional hazard. Am J Clin Nutr.

[32] Dagba B, Sambe L (2013). Totemic Beliefs and Biodiversity Conservation among the Tiv People of Benue State. Nigeria Journal of Natural Sciences Research.

[33] Sharifah Zahhura SA, Nilan P, Germov J (2012). Food restrictions during pregnancy among Indigenous Temiar women in peninsular Malaysia. Malaysian journal of nutrition.

[34] Manyande A, Grabowska C (2009). Factors affecting the success of moxibustion in the management of a breech presentation as a preliminary treatment to external cephalic version. Midwifery.

